# *KIF23* silencing suppresses papillary thyroid carcinoma metastasis by regulating mitophagy via Wnt/β-catenin pathway

**DOI:** 10.1530/EC-25-0090

**Published:** 2025-10-24

**Authors:** Ying Liu, Junping Zhang, Yuhang Chen, Mengya Zhu, Wen Chen, Zejin Hao, Yuanyuan Deng, Xiudan Han, Jixiong Xu

**Affiliations:** ^1^ Department of Endocrine and Metabolism, The First Affiliated Hospital, Jiangxi Medical College, Nanchang University, Nanchang, China; ^2^ Jiangxi Clinical Research Center for Endocrine and Metabolic Disease, Nanchang, Jiangxi, China; ^3^ Jiangxi Branch of National Clinical Research Center for Metabolic Disease, Nanchang, Jiangxi, China

**Keywords:** *KIF23*, Wnt/β-catenin, mitophagy, papillary thyroid carcinoma

## Abstract

**Background:**

Kinesin family member 23 (KIF23) plays a critical role in the regulation of cell division. This study aims to explore the function of KIF23 and its underlying regulatory mechanisms in the progression of papillary thyroid carcinoma (PTC).

**Methods:**

*KIF23* expression was analyzed in PTC and adjacent tissues using RNA sequencing (RNA-Seq) data in The Cancer Genome Atlas (TCGA). Immunohistochemistry (IHC) and quantitative reverse transcription PCR (qRT-PCR) were performed to assess *KIF23* expression in PTC tissues and cell lines. A *KIF23* knockdown cell line was established to evaluate its effects on cell proliferation and migration via cell counting kit-8 (CCK-8), colony formation, Transwell migration, and wound-healing assays. Western blotting (WB) was used to analyze Wnt/β-catenin signaling and mitophagy markers.

**Results:**

*KIF23* transcript levels were significantly elevated in PTC tissues compared to adjacent normal tissues, correlating with poor progression-free interval. IHC staining confirmed the upregulation of KIF23 in PTC tissues, while qRT-PCR analysis verified the increased mRNA expression of *KIF23* in cell lines. *KIF23* knockdown reduced cell proliferation, migration, and invasion and decreased levels of Wnt/β-catenin signaling proteins β-catenin (*CTNNB1*) and c-Myc (*MYC*) while increasing mitophagy markers Parkin (*PRKN*), PTEN-induced kinase 1 (PINK1), and LC3B (*MAP1LC3B*). Wnt agonist treatment reversed these effects, and both the Wnt agonist and the mitophagy inhibitor Mdivi-1 were able to rescue the migratory inhibition caused by *KIF23* knockdown.

**Conclusions:**

KIF23 regulates mitophagy via the Wnt/β-catenin pathway, influencing PTC cell proliferation and migration, suggesting its potential as a therapeutic target for PTC.

## Introduction

Thyroid carcinoma (THCA) is the most prevalent endocrine malignancy, with its incidence steadily increasing worldwide (1). According to the Global Cancer Statistics 2020, there were 586,000 new cases of THCA, making it the 9th most common cancer overall and the 5th most common in women (2). Papillary thyroid carcinoma (PTC), the most prevalent subtype of THCA, is characterized by high differentiation and relatively indolent progression (3, 4). Although the prognosis for PTC is generally favorable, the risk of cervical lymph node metastasis (LNM) remains significant and serves as a major predictor of recurrence and distant metastasis (5). Currently, diagnostic imaging techniques, such as ultrasound and computed tomography, exhibit a sensitivity of only 25–60% in detecting cervical LNM in PTC patients (6), making the early identification of high-risk individuals a considerable challenge. The main treatment modalities for PTC include surgical resection, thyroid-stimulating hormone suppression therapy, and radioactive iodine (^131I) therapy. However, despite these interventions, approximately 41,000 deaths annually are still attributed to the high rates of recurrence and metastasis associated with PTC (7, 8). This underscores the urgent need for reliable biological markers that can predict poor prognosis in PTC patients. Identifying such markers is critical for improving diagnostic accuracy and tailoring more effective treatment strategies.

The kinesin superfamily (KIF) is a group of motor proteins primarily found in eukaryotic cells, playing essential roles in intracellular transport, cell division, and microtubule dynamics (9, 10). Among them, Kinesin family member 23 (KIF23) is particularly important for maintaining proper cell function by regulating these processes. Dysregulation of *KIF23* expression can disrupt cell division and impair critical cellular processes, ultimately affecting cell growth and survival (11). In recent years, abnormal *KIF23* expression has been linked to the initiation and progression of various cancers. For instance, overexpression of *KIF23* was correlated with tumor invasiveness and poor prognosis of hepatocellular carcinoma (12). Similarly, in breast cancer, KIF23 promotes tumor cell proliferation and migration through the Wnt/β-catenin signaling pathway (13). In addition, *KIF23* expression may influence the prognosis of nasopharyngeal carcinoma by regulating mitophagy (14). Although these findings underscore the pivotal role of in cancer biology, its specific function in PTC remains unclear.

In this study, we found that *KIF23* expression is upregulated in PTC cells and tissues, and its increased expression was strongly correlated with a shorter progression-free interval (PFI). Functional experiments further showed that knockdown of *KIF23* significantly inhibited the cellular malignant phenotype (including cell proliferation and migration) of PTC cells. In addition, our study revealed that KIF23 may regulate mitophagy through the Wnt/β-catenin signaling pathway, which in turn affects cell migration. These results suggest that KIF23 may play an important role in the development of PTC and provide a theoretical basis for exploring new targeted therapeutic strategies.

## Materials and methods

### Expression, prognosis, and gene set enrichment analysis (GSEA)

We analyzed 505 PTC samples and 59 adjacent normal tissue samples obtained from The Cancer Genome Atlas (TCGA) (https://portal.gdc.cancer.gov/). RNA sequencing (RNA-Seq) data and corresponding clinical information were retrieved for all samples. Differential expression analysis of *KIF23* between PTC and adjacent normal tissues was performed using the TCGA-THCA dataset. Genes with an adjusted *P*-value <0.05 were considered to be differentially expressed. Based on the median expression level of *KIF23*, the samples were categorized into high-expression and low-expression groups. Kaplan–Meier (KM) survival curves were then generated to assess differences in PFI between these two groups. GSEA was performed using GSEA_Linux_4.2.3 software. The reference gene set, c2.cp.v2023.1.Hs.symbols.gmt, was obtained from the Molecular Signatures Database (MSigDB) (https://www.gsea-msigdb.org/gsea/msigdb/download_file.jsp?filePath=/msigdb/release/2023.1.Hs/c2.cp.v2023.1.Hs.symbols.gmt). Statistical significance was defined according to the standard GSEA criteria, namely an adjusted *P*-value <0.05 and a false discovery rate (FDR) value < 0.25, with *P*-value correction performed using the Benjamini–Hochberg (BH) method (15).

### Patient tissue samples

Tissue samples were collected from 15 patients with PTC who underwent surgical resection at the First Affiliated Hospital of Nanchang University between June 2023 and July 2024 for immunohistochemical analysis. All samples were confirmed by pathological examination.

### Cells and cell culture

The human thyroid normal epithelial cell line Nthy-ori-3-1 (CL-0817) was purchased from Procell Biotechnology, Wuhan, China. The human PTC cell line, TPC-1 (ml097683), was purchased from Mlbio Biotechnology, Shanghai, China. The K1 (FH1196) and BCPAP (FH1046) cell lines were obtained from Fuheng Biotechnology, Shanghai, China. Nthy-ori-3-1, TPC-1, and BCPAP cells were cultured in Roswell Park Memorial Institute (RPMI) 1,640 medium supplemented with 10% fetal bovine serum (FBS, Thermo Fisher Scientific, USA). K1 cells were cultured in high-glucose Dulbecco’s modified Eagle medium (DMEM) containing 10% FBS. All cells were maintained at 37°C in a humidified atmosphere with 5% CO_2_. All cell lines used in this study were authenticated by short tandem repeat profiling and were regularly tested for mycoplasma contamination to confirm their identity and to prevent any misidentification or contamination.

### Quantitative reverse transcription PCR (qRT-PCR)

Total RNA was extracted from cultured cells using TransZol reagent (ER501-01, TransGen Biotech, China) according to the manufacturer’s instructions. RNA concentration and purity were measured using a NanoDrop 2000 spectrophotometer (Thermo Fisher Scientific, USA). RNA integrity was evaluated by 1% agarose gel electrophoresis, which revealed distinct 28 and 18S ribosomal RNA bands (Supplementary Fig. 1 (see section on Supplementary materials given at the end of the article)). cDNA was synthesized from 1 μg of total RNA using HiScript III RT SuperMix for qPCR (+gDNA Wiper) (R323-01, Vazyme, China). Quantitative PCR (qPCR) was performed on a CFX96 Real-Time PCR Detection System (Bio-Rad, USA) using AceQ qPCR SYBR Green Master Mix (Q111-02, Vazyme, China). Cycling conditions were: 95°C for 5 min, followed by 40 cycles of 95°C for 10 s and 60°C for 30 s, with a final melt curve analysis to confirm specificity. Gene expression levels were calculated using the 2^(−ΔΔCt) method. GAPDH served as the internal control. Each sample was analyzed in triplicate technical replicates, and three independent biological replicates were performed. Primer sequences are listed in Supplementary Table 1.

### RNA interference and transfection protocol

The lentiviral KIF23 plasmid and the control empty vector were purchased from Qingke Biotechnology Company (China). The specific sequences used are provided in Supplementary Table 2. For cell transfection, cells were passaged and digested with 0.25% trypsin (Thermo Fisher Scientific, USA) to generate a single-cell suspension, which was subsequently seeded into a 6-well plate and cultured at 37°C with 5% CO_2_. Cells were grown until they reached approximately 50–70% confluence. After 24 h, when the cells were in optimal condition, the culture medium was removed, and the infection solution was added. The viral solution, containing lentivirus at the appropriate multiplicity of infection, was added to each well. Following infection, cells were incubated for 18–20 h, after which the medium was replaced with fresh culture medium. Transfection efficiency was assessed by fluorescence microscopy (Olympus, Japan) 72 h post-infection; infection was considered successful when >80% of cells exhibited green fluorescent protein (GFP) fluorescence without overcrowding or morphological abnormalities. Selection of stable transfectants was initiated by adding puromycin (Thermo Fisher Scientific, USA) to the culture medium at a final concentration of 2 ng/mL for 48 h. Only cultures maintaining >80% viability under selection were expanded for subsequent experiments. Successful knockdown was further validated by qRT-PCR and western blotting.

### Immunohistochemistry (IHC)

PTC tissue and adjacent normal tissue located more than 2 cm from the cancer were collected and fixed in 4% formaldehyde for 48 h, followed by paraffin embedding and sectioning. The sections were then heated at 60°C for 30 min, followed by deparaffinization and rehydration using xylene and ethanol at varying concentrations. Next, the tissue sections were subjected to antigen retrieval by microwaving in citrate buffer, and endogenous peroxidase activity was blocked using 3% hydrogen peroxide solution. The sections were incubated overnight at 4°C with anti-rabbit KIF23 antibody (DF2573, 1:100, Affinity, USA). After incubation with the secondary antibody, the sections were stained with DAB substrate solution and observed under a microscope for imaging. Finally, the staining intensity was quantified using ImageJ software.

### Cell counting kit-8 (CCK-8) assay

The transfected cells were collected using trypsin digestion, counted, and then seeded into 96-well cell culture plates at a density of 4 × 10^3^ cells per well. The plates were incubated overnight at 37°C in a 5% CO_2_ incubator. After culturing at 37°C for 24, 48, 72, and 96 h, 10 μL of fresh medium containing 10% CCK-8 reagent (Sigma, USA) was added to each well and incubated for an additional 2 h. The optical density values were then measured at 450 nm using a microplate reader. All observations were repeated at least three times in independent experiments, with each experiment including more than three technical replicates.

### Clone formation assay

Transfected TPC-1 and K1 cells, along with their respective control group cells, were seeded into 6-well plates according to their groups, with approximately 500 cells per well. During the culture period, the medium was replaced every 3 days, and the cells were cultured for 2 weeks. Following this, the cells were fixed with 4% paraformaldehyde (Beyotime, China) for 30–60 min, and then washed once with PBS. Next, 500 μL of GIEMSA staining solution (Beyotime, China) was added to each well for staining, with a staining time of 10–20 min. After staining, the cells were washed again with PBS and allowed to air dry. Finally, the plates were photographed, and the number of clones with more than 10 cells per well was counted and recorded.

### Wound healing assay

TPC-1 and K1 cells were seeded at 5 × 10^5^ cells per well in 6-well plates. Once the cells reached 80% confluence, they were gently mixed using the figure-eight method. A sterile 200 μL pipette tip was then used to create a vertical scratch in the cell monolayer. The medium was aspirated, and the wells were washed three times with PBS to remove the detached cells. The medium was subsequently replaced with 1% FBS culture medium, and the remaining cells were incubated for an additional 24 h. Images were captured at 0 and 24 h post-scratch using an inverted microscope.

### Transwell invasion and migration assay

Cells were harvested by trypsin digestion, followed by centrifugation. The supernatant was discarded, and the cells were resuspended in serum-free medium and counted. For the invasion assay, Matrigel (BioFroxx, Germany) was thawed overnight at 4°C and kept on ice. It was then diluted with ice-cold serum-free medium at a 1:3 ratio. Next, 50 μL of the diluted Matrigel was carefully added to the upper chamber of the Transwell insert and incubated at 37°C for 2–4 h to allow gel formation. For the migration assay, no Matrigel coating was applied. Once the chambers were prepared, 200 μL of cell suspension containing 5 × 10^4^ cells (for invasion) or 1 × 10^5^ cells (for migration) in serum-free medium was added to each upper chamber. The distribution of cells was confirmed under a microscope. Afterward, 600 μL of complete medium containing 5% FBS was added to the lower chamber as a chemoattractant. The plate was incubated at 37°C with 5% CO_2_ for 12–24 h. After the incubation period, non-migrated cells on the upper surface of the membrane were removed using a cotton swab. The Transwell inserts were then fixed in 4% paraformaldehyde for 30 min at room temperature, washed twice with PBS, and stained with 0.1% crystal violet for 20 min. Excess dye was removed by rinsing with PBS. The migrated cells on the lower membrane surface were counted in five randomly selected fields under 100× magnification. The average number of cells per field was calculated for quantitative analysis.

### Western blot (WB) analysis

Total protein was extracted from cells using RIPA lysis buffer (GLPBIO, USA) supplemented with protease and phosphatase inhibitors (GLPBIO, USA). Proteins were separated by SDS-PAGE and transferred onto PVDF membranes (Millipore, Germany). The membranes were blocked with 5% nonfat dry milk in PBS containing 0.1% Tween 20 (PBST) for 1 h, followed by incubation with diluted primary antibody: rabbit anti-KIF23 (DF2573, 1:1,000, Affinity, USA), mouse anti-GAPDH (60004-1-1g, 1:30,000, Proteintech, USA), rabbit anti-c-Myc (10828-1-AP, 1:12,000, Proteintech, USA), mouse anti-Parkin (4211S, 1:1,000, CST, USA), rabbit anti-P62 (39749S, 1:1,000, CST, USA), mouse anti-PINK1 (ab186303, 1:1,000, Abcam, UK), rabbit anti-LC3B (AF4650, 1:1,000, Affinity, USA), overnight at 4°C. After incubation, the membranes were washed three times with PBST for 5 min each, then incubated with horseradish peroxidase-conjugated secondary antibody (A0208, A0216, Beyotime Biotech, China) for 2 h at room temperature, followed by three washes with TBST. Protein bands were visualized using ECL (TIANGEN Biotech, China) and quantified with ImageJ software using GAPDH as the internal control. Each experiment was repeated at least three times.

### Statistical analysis

Data analysis was performed using SPSS 22.0, GraphPad Prism 8.0, and ImageJ software. Quantitative data are presented as mean ± standard deviation (SD). For comparisons between two groups, an independent-sample *t*-test was applied when data followed a normal distribution with homogeneous variance. For comparisons among more than two groups, one-way ANOVA was used under the same assumptions. When data exhibited skewed distributions or variance heterogeneity (such as in functional assays, qPCR experiments, and grayscale analysis), we employed corrected versions of the parametric tests – specifically Welch’s *t*-test for two-group comparisons and Welch’s/Brown–Forsythe one-way ANOVA for multiple groups – which are robust against violations of normality and homogeneity of variance. Each experiment was independently repeated at least three times to ensure reliability. A *P* value less than 0.05 was considered statistically significant.

## Results

### High expression of *KIF23* in PTC

**Figure 1:**
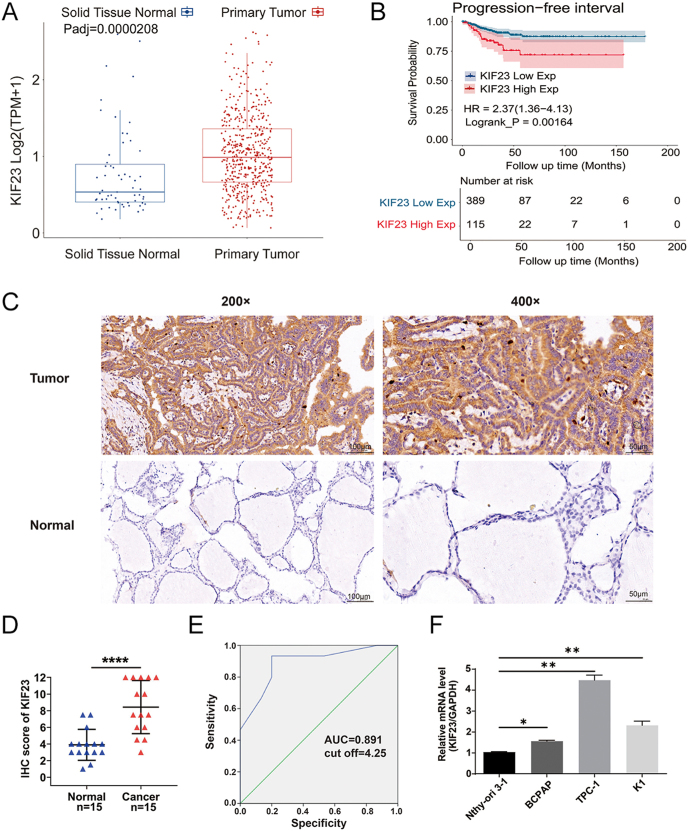
Validation of *KIF23* expression in PTC. (A) Differential expression analysis of *KIF23* between PTC (*n* = 505) and adjacent normal tissues (*n* = 59) using RNA-seq data from the TCGA-THCA dataset. (B) Kaplan–Meier (KM) survival analysis evaluating the association between *KIF23* expression and PFI in PTC patients using RNA-seq data from the TCGA-THCA dataset. (C) Representative images of IHC staining of KIF23 protein (50 and 100 μm scale bar) in PTC and paired adjacent normal tissues. (D) IHC staining scores revealed that KIF23 levels were upregulated in PTC tissues (*n* = 15). (E) Receiver operating characteristic (ROC) curve analysis determined that the optimal diagnostic cut-off value of IHC score was 4.25 and the area under the curve (AUC) was 0.891. (F) qRT-PCR detection of *KIF23* expression in each PTC cell line. **P < 0.01, ***P < 0.001, ****P < 0.0001.

To investigate the potential role of KIF23 in PTC, we began by conducting bioinformatics analysis using the TCGA-THCA dataset, which includes RNA-Seq data from both PTC tissues and adjacent normal thyroid tissues. Our analysis revealed that *KIF23* expression was significantly upregulated in PTC tissues compared with normal tissues (*P* < 0.0001) (Fig. 1A). Based on the median *KIF23* expression level in the TCGA-THCA cohort, patients were stratified into high- and low-expression groups. Kaplan–Meier survival analysis demonstrated that elevated *KIF23* expression was significantly associated with shorter PFI in PTC patients (*P* < 0.01, HR = 2.37, 95% CI: 1.36–4.13) (Fig. 1B). To validate these findings at the protein level, IHC staining was performed on 15 pairs of patient-derived tissue samples from PTC tumors and adjacent normal thyroid tissues. The IHC results demonstrated markedly higher KIF23 levels in tumor tissues compared with adjacent normal tissues (Fig. 1C and D), confirming the upregulation of KIF23 in PTC. Moreover, ROC curve analysis of the IHC scores identified an optimal diagnostic cutoff value of 4.25, yielding a sensitivity of 93.3%, specificity of 80.0%, and an area under the curve (AUC) of 0.891, suggesting the potential diagnostic value of KIF23 in PTC (Fig. 1E). In addition, qRT-PCR was performed to assess *KIF23* expression in PTC cell lines (BCPAP, K1, and TPC-1) as well as in the normal thyroid cell line Nthy-ori-3-1. Consistent with the tissue-level findings, *KIF23* expression was significantly elevated in all three PTC cell lines relative to the normal thyroid cells (Fig. 1F). Taken together, these results strongly suggest that KIF23 plays a crucial role in the development and progression of PTC.

### Construction of knockdown cell model and the effect of *KIF23* knockdown on cell proliferation

**Figure 2:**
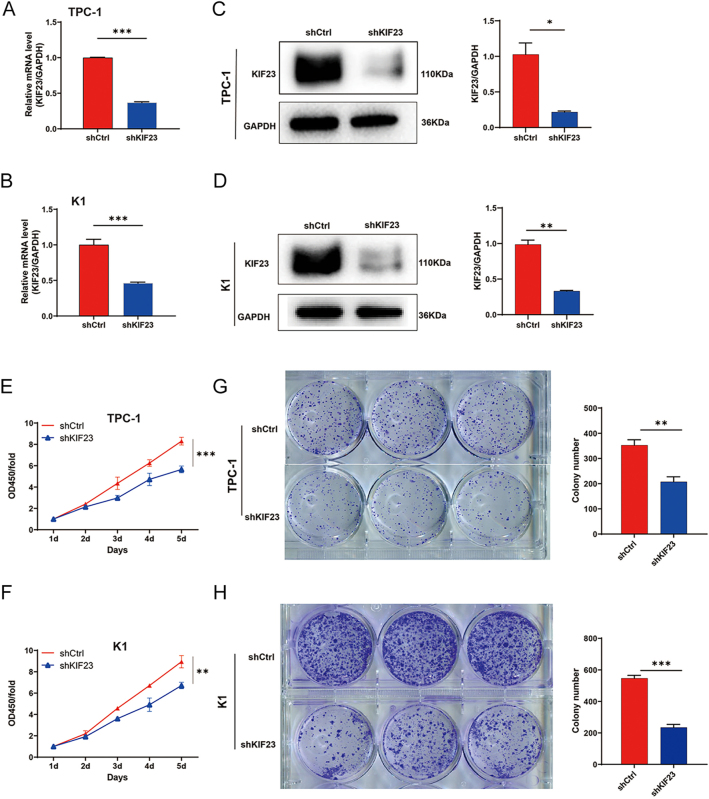
Validation of *KIF23* knockdown efficiency. (A) qRT-PCR analysis showing the efficiency of *KIF23* knockdown in TPC-1 cells. (B) qRT-PCR analysis demonstrating the efficiency of *KIF23* knockdown in K1 cells. (C) WB analysis confirming that *KIF23* knockdown significantly reduced KIF23 protein levels in TPC-1 cells. (D) WB analysis showing a marked reduction in KIF23 protein levels following *KIF23* knockdown in K1 cells. (E) CCK-8 assay assessing cell viability in the TPC-1 cell lines from each experimental group. (F) CCK-8 assay evaluating cell viability of each group of K1 cells. (G) Clone formation assay measuring the proliferative ability of TPC-1 cells in each treatment group. (H) Clone formation assay assessing the proliferative potential of K1 cells in each experimental group. Compared with the shCtrl group, *P < 0.05, **P < 0.01, ***P < 0.001.

To investigate the effects of *KIF23* on PTC *in vitro*, we generated knockdown cell models using lentiviral vectors targeting *KIF23* in TPC-1 cells. Compared to the *shCtrl* group, the *KIF23* mRNA knockdown efficiency in the *shKIF23-3* group was 91.6%, significantly higher than observed in the *shKIF23-1* and *shKIF23-2* groups. Therefore, the *shKIF23-3* lentivirus was selected for further experiments (Supplementary Fig. 2A). Fluorescence microscopy revealed that the infection efficiency in both the *shCtrl* and *shKIF23* groups exceeded 80%, with normal cell morphology observed, confirming successful transfection (Supplementary Fig. 2B). qRT-PCR analysis revealed knockdown efficiencies of 63.0% in TPC-1 cells and 53.9% in K1 cells in the *shKIF23* group (Fig. 2A and B). In addition, WB analysis demonstrated a significant reduction in KIF23 protein levels in the *shKIF23* group, with a decrease of 45.2% in TPC-1 cells and 56.7% in K1 cells (Fig. 2C and D). These results collectively confirm the successful establishment of *KIF23* knockdown cell models.

We then assessed the effects of *KIF23* knockdown on cell proliferation using CCK-8 and colony formation assays. Both TPC-1 and K1 cells infected with the *KIF23* knockdown lentivirus exhibited a significant decrease in cell viability (Fig. 2E and F). Colony formation assays further revealed that *KIF23* knockdown markedly inhibited the proliferation of both TPC-1 and K1 cells (Fig. 2G and H). These findings indicate that *KIF23* knockdown effectively suppresses the proliferation of PTC cells.

**Figure 3:**
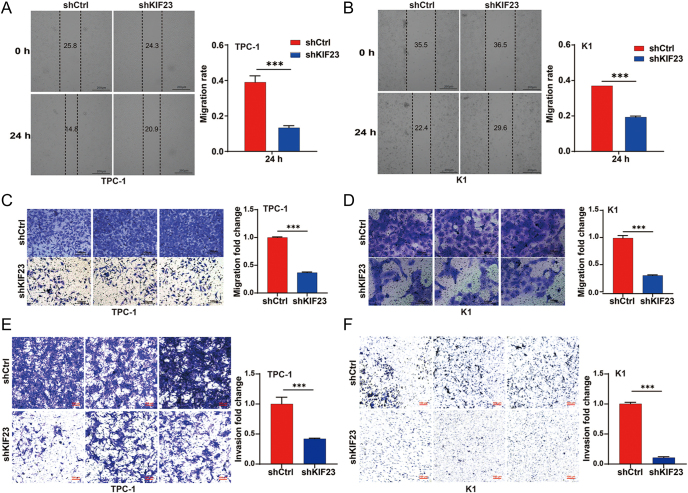
Knockdown of *KIF23* inhibits migration and invasion of PTC cells. (A and B) Scratch wound assay performed to assess the migration capacity of each group in TPC-1 cells (A) and K1 cells (B). (C and D) Transwell migration assay used to evaluate the migration ability of each group in TPC-1 cells (C) and K1 cells (D). (E and F) Transwell invasion assay conducted to examine the invasive potential of each group in TPC-1 cells (E) and K1 cells (F). Compared with the shCtrl group, ***P < 0.001.

In addition, we evaluated the impact of *KIF23* knockdown on cell migration and invasion using scratch wound, Transwell migration, and Transwell invasion assays. The scratch wound assay showed a notable decrease in the migration rate of both TPC-1 and K1 cells following *KIF23* silencing (Fig. 3A and B). The Transwell migration assay further confirmed that KIF23 silencing led to a significant reduction in the migration capacity of PTC cells (Fig. 3C and D). Furthermore, the Transwell invasion assay showed a marked inhibition of the invasive potential of PTC cells upon *KIF23* knockdown, reinforcing the critical role of *KIF23* in regulating both migration and invasion (Fig. 3E and F). These findings collectively suggest that silencing *KIF23* effectively suppresses both the migratory and invasive abilities of PTC cells.

### KIF23 mediates PTC cell migration via the Wnt/β-catenin pathway

**Figure 4:**
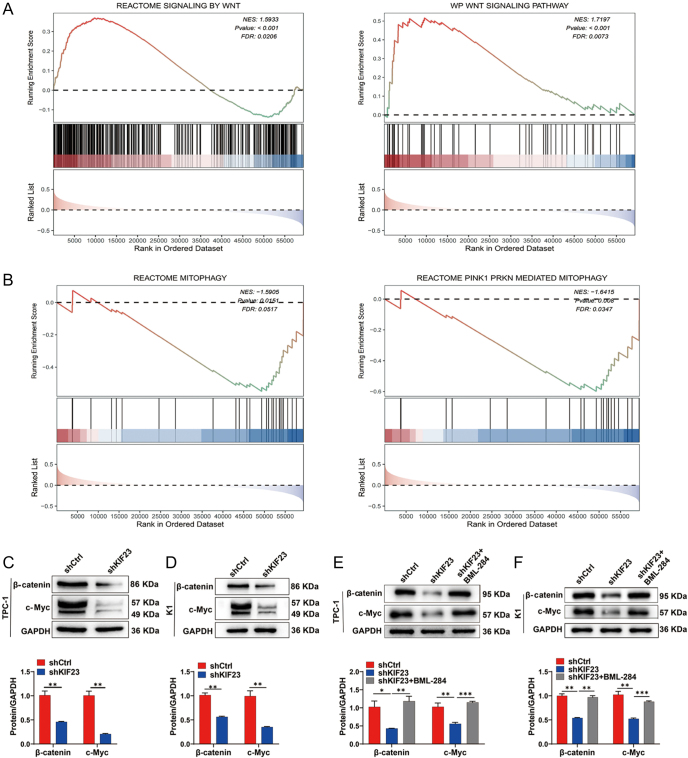
KIF23 mediates the migration of PTC cells through the Wnt/β-catenin pathway. (A) GSEA showing significant enrichment of the Wnt signaling pathway in the high *KIF23* expression group based on NES values. (B) GSEA showing significant enrichment of mitophagy components in the low *KIF23* expression group based on NES values. The NES value represents the enrichment score after normalization, with higher values indicating greater gene enrichment in the pathway. Significant enrichment was determined using thresholds of *P*.adj <0.05 and FDR <0.10. (C) WB used to detect the levels of β-catenin and c-Myc in each group of the TPC-1 cell line. (D) WB was used to detect the levels of β-catenin and c-Myc in each group of the K1 cell line. (E) WB analysis performed to detect the levels of β-catenin and c-Myc in each group of the TPC-1 cell line after *KIF23* knockdown co-treatment with the WNT agonist BML-284. (F) WB analysis performed to detect the levels of β-catenin and c-Myc in each group of the K1 cell line after *KIF23* knockdown co-treatment with the Wnt agonist BML-284. Compared with the control group, *P < 0.05, **P < 0.01, ***P < 0.001.

To investigate the molecular mechanisms underlying the role of KIF23 in PTC progression, we conducted GSEA using RNA sequencing data from TCGA-THCA samples. The GSEA results based on *KIF23* expression revealed that the Wnt signaling pathway and mitophagy components were significantly enriched in the TCGA-THCA dataset (FDR <0.10 and *P*-value <0.05) (Fig. 4A and B). To further examine whether the Wnt/β-catenin signaling pathway is involved in KIF23-mediated PTC progression, we evaluated the levels of β-catenin (encoded by *CTNNB1*) and its downstream target, c-Myc (encoded by *MYC*), in PTC cells upon *KIF23* knockdown. Western blot analysis revealed that knockdown of *KIF23* significantly reduced the levels of β-catenin and c-Myc in both TPC-1 and K1 cells (Fig. 4C and D). Importantly, the addition of the Wnt agonist BML-284 to the shKIF23 group resulted in a significant increase in the levels of β-catenin and c-Myc in both cell lines, suggesting that the Wnt agonist effectively counteracted the inhibitory effects of *KIF23* knockdown (Fig. 4E and F).

**Figure 5:**
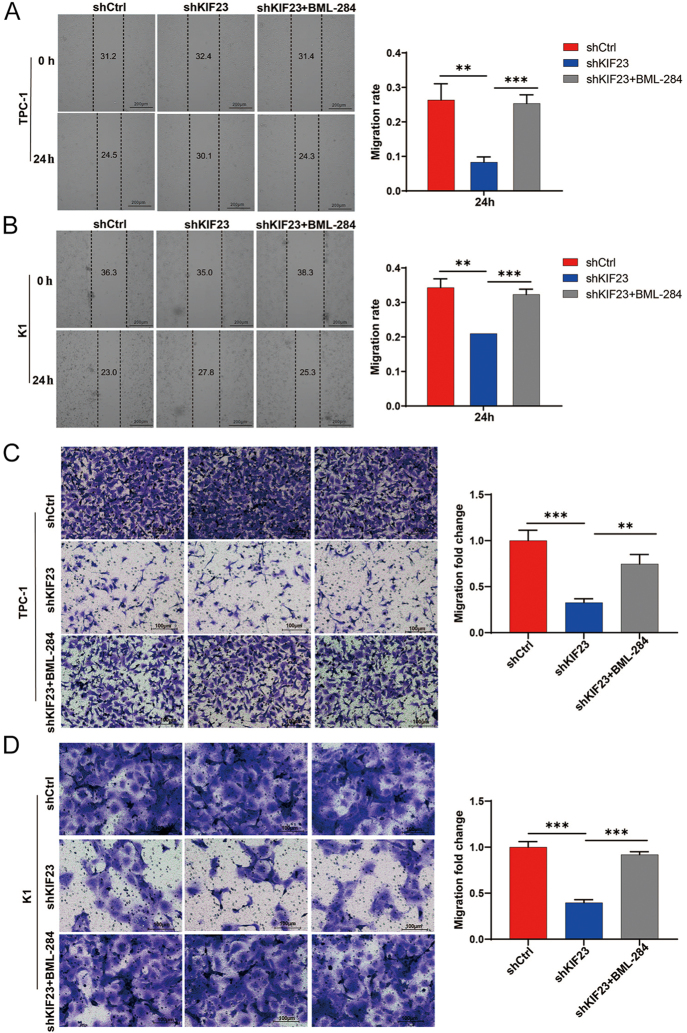
KIF23 promotes migration of PTC cells through the Wnt/β-catenin pathway. (A) Scratch wound assay assessing the migration capacity of TPC-1 cells in each experimental group. Knockdown of *KIF23* enhanced the migration ability of TPC-1 cells, which was partially reversed by treatment with the Wnt agonist BML-284. (B) Scratch wound assay evaluating the migration ability of K1 cells in the different experimental groups. Similar to TPC-1 cells, *KIF23* knockdown increased migration, which was partially inhibited by BML-284. (C) Transwell migration assay measuring the migratory potential of TPC-1 cells in each group. *KIF23* knockdown significantly enhanced migration, which was attenuated upon BML-284 treatment. (D) Transwell migration assay assessing cell migration in K1 cells. Knockdown of *KIF23* increased migration, and this effect was partially reversed by the Wnt agonist BML-284. Compared with the control group, *P < 0.05, **P < 0.01, ***P < 0.001.

Subsequently, we assessed the impact of the Wnt agonist on cell migration using both scratch wound and Transwell assays. The results indicated that knockdown of *KIF23* significantly enhanced the migratory capacity of PTC cells. This effect was partially reversed by treatment with BML-284, as shown in both the scratch assay (Fig. 5A and B) and Transwell assay (Fig. 5C and D). These findings indicate that the Wnt agonist effectively reversed the inhibitory effects of *KIF23* knockdown on cell migration. Taken together, our data suggest that *KIF23* knockdown inhibits PTC cell migration by downregulating the Wnt/β-catenin signaling pathway.

### KIF23 regulates mitophagy via the Wnt/β-catenin pathway

**Figure 6:**
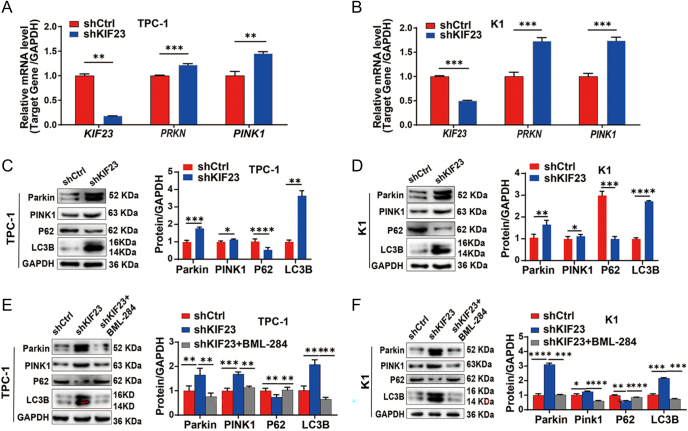
KIF23 regulates mitophagy in PTC cells (TPC-1 and K1). (A) qRT-PCR to detect the mRNA expression of *KIF23* (encoding KIF23), *PRKN* (encoding Parkin), and *PINK1* (encoding PINK1), in each group in the TPC-1 cell line. (B) qRT-PCR to detect the mRNA expression of *KIF23*, *PRKN,* and *PINK1* in each group in the K1 cell line. (C) WB to detect the protein levels of Parkin, PINK1, P62, and LC3B in each group in the TPC-1 cell line. (D) WB to detect the protein levels of Parkin, PINK1, P62, and LC3B in each group in the K1 cell line. (E) WB detection of Parkin, PINK1, P62, and LC3B levels after addition of Wnt agonist BML-284 in the TPC-1 cell line. (F) WB detection of Parkin, PINK1, P62, and LC3B levels after addition of Wnt agonist BML-284 in the K1 cell line. Compared with the control group, *P < 0.05, **P < 0.01, ***P < 0.001, ****P < 0.0001.

As shown in Fig. 4A and B, GSEA based on *KIF23* expression revealed significant enrichment in both the Wnt signaling pathway and mitophagy (FDR <0.10 and *P*-value <0.05). Building on these results, we further investigated the potential role of KIF23 in regulating mitophagy in PTC cells. QRT-PCR and WB analysis indicate that the expression of mitophagy markers Parkin (encoded by *PRKN*) and PTEN-induced kinase 1 (PINK1) (encoded by *PINK1*) was significantly upregulated at both the RNA and protein levels in the *shKIF23* group. In addition, the level of LC3B (encoded by *MAP1LC3B*) was significantly elevated, whereas the level of P62 (encoded by *SQSTM1*) was notably reduced (Fig. 6A, B, C, D). These findings suggest that *KIF23* knockdown significantly enhances the expression of mitophagy-related genes, highlighting its potential role in regulating mitophagy in PTC cells.

To investigate whether KIF23 regulates mitophagy through the Wnt/β-catenin signaling pathway, we treated the *shKIF23* cells with the Wnt agonist BML-284. Notably, co-treatment with BML-284 reversed the upregulation of *PRKN* and *PINK1* mRNA expression (Supplementary Fig. 3). Meanwhile, WB analysis showed that the increase in the protein levels of Parkin, PINK1, and LC3B, as well as the decrease in P62, following *KIF23* knockdown, could be partially reversed by the addition of BML-284 (Fig. 6E and F). These findings suggest that Wnt agonist treatment reversed the increase in mitophagy marker expression caused by *KIF23* knockdown, indicating that KIF23 regulates mitophagy in PTC cells through the Wnt/β-catenin pathway.

### KIF23 regulates mitophagy to mediate migration in PTC

**Figure 7:**
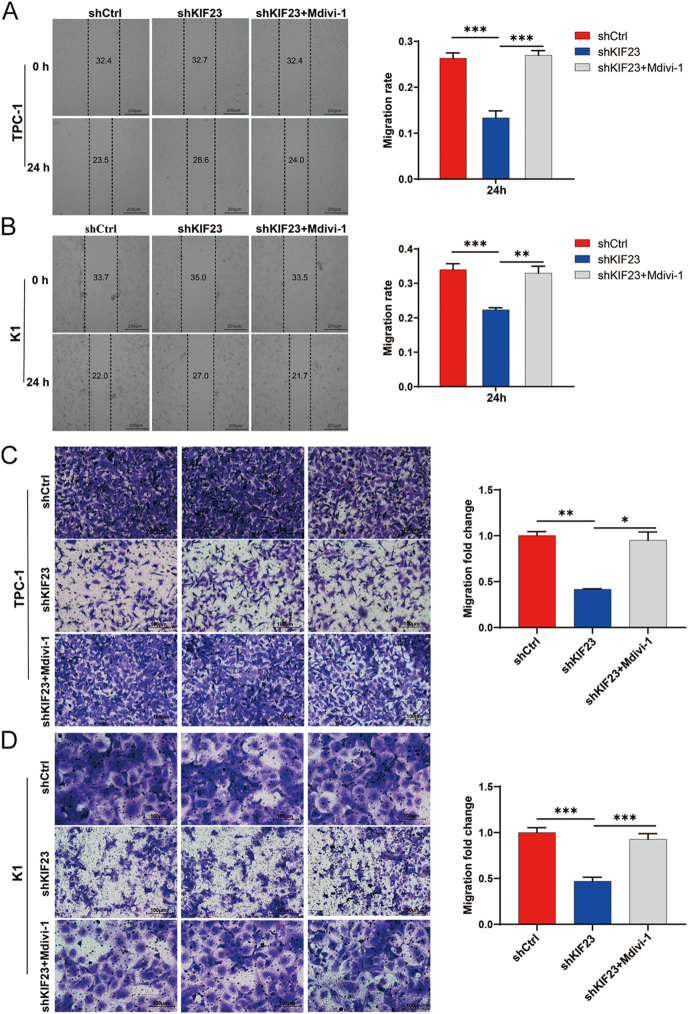
KIF23 regulates mitophagy-mediated cell migration in PTC cells (TPC-1 and K1). (A and B) Scratch wound assay assessing cell migration levels in PTC cells from each experimental group. Inhibition of mitophagy by the mitophagy inhibitor Mdivi-1 partially restored the migratory ability of TPC-1 cells (A) and K1 cells (B) following *KIF23* knockdown. (C and D) Transwell migration assay measuring migration levels in PTC cells from each experimental group. Mitophagy inhibitor Mdivi-1 treatment partially restored the migration ability of TPC-1 cells (C) and K1 cells (D) after *KIF23* knockdown. Compared with the control group, *P < 0.05, **P < 0.01, ***P < 0.001.

To explore whether KIF23 regulates PTC cell migration through mitophagy, we treated the *KIF23* knockdown group with the mitophagy inhibitor Mdivi-1 (*shKIF23* + Mdivi-1). The results from the scratch wound assay indicated that the migration rate in the *shKIF23* + Mdivi-1 group was significantly higher compared to the *shKIF23* group (Fig. 7A and B). Similarly, the results from the Transwell assay confirmed these findings, showing that the migration rate in the *shKIF23* + Mdivi-1 group was remarkably greater than in the *shKIF23* group (Fig. 7C and D). These results suggest that the inhibition of mitophagy by Mdivi-1 partly restores the migratory ability of PTC cells following *KIF23* knockdown. Taken together, these findings imply that KIF23 may regulate PTC cell migration by modulating mitophagy.

## Discussion

In this study, we found that *KIF23* was highly expressed in PTC, and its downregulation significantly inhibited the proliferation and migration of PTC cells. Furthermore, we discovered that silencing *KIF23* led to a downregulation of proteins associated with the Wnt/β-catenin signaling pathway, along with an upregulation of mitophagy-related proteins. Interestingly, these molecular alterations were partially reversed by the addition of a Wnt agonist. Moreover, the impaired migratory ability of PTC cells with *KIF23* knockdown was restored upon treatment with a mitophagy inhibitor. These insights into the molecular mechanisms of KIF23 provide a promising foundation for developing targeted therapies against PTC.

KIF23, a kinesin family protein, plays a crucial role in cell division, cycle regulation, and mitosis by participating in key steps of these processes (16, 17). In recent years, the dysregulation of *KIF23* expression was found in the development and progression of various human cancers (18). For instance, studies have demonstrated that the elevated *KIF23* expression in colorectal cancer and pancreatic ductal adenocarcinoma is significantly correlated with shorter overall survival and poorer prognosis (19, 20). Consistent with previous studies, our findings indicate that the high expression of *KIF23* is strongly linked to a decrease in PFI in PTC patients. Furthermore, overexpression of *KIF23* has been shown to promote malignant phenotypes by accelerating cell proliferation and inhibiting pyroptosis, thereby facilitating tumor progression of cervical cancer (21). In addition, in breast cancer, the upregulation of *KIF23* has been shown to enhance cancer cell proliferation and promote malignant behavior, potentially mediated through the Wnt/β-catenin signaling pathway (13, 22). Our results further support previous studies showing that downregulation of *KIF23* significantly inhibits the proliferation and migration of TPC-1 and K1 cells, indicating that KIF23 may be involved in the onset and development of PTC. In addition, GSEA based on *KIF23* expression revealed significant enrichment of the Wnt signaling pathway in the TCGA-THCA dataset. This observation leads us to hypothesize that KIF23 may influence PTC development and progression through the modulation of this pathway.

The Wnt/β-catenin signaling pathway is an evolutionarily conserved pathway that plays a critical role in embryonic development and the maintenance of tissue homeostasis (23). Dysregulation of the Wnt/β-catenin signaling pathway is implicated in the development and progression of several serious diseases, including a wide range of malignant tumors. For example, Tang *et al.* (24) demonstrated that TM4SF1 maintains tumor stemness and promotes epithelial–mesenchymal transition (EMT) by upregulating Wnt/β-catenin signaling, thereby increasing the recurrence and metastasis rates in colorectal cancer. Similarly, the role of the Wnt/β-catenin signaling pathway in gastric cancer has been demonstrated by a study showing that SERPINH1 promotes the proliferation and metastasis of gastric cancer cells by activating this pathway (25). As for the regulation of PTC progression by Wnt/β-catenin signaling, one study demonstrated that CDK5RAP3 inhibits Wnt/β-catenin signaling by downregulating the Akt/GSK-3β pathway, thereby reducing the proliferation, migration, and invasion of PTC cells (26), while another study revealed that ASPM promotes the proliferation, migration, and invasion of undifferentiated PTC cells *in vitro* by activating the Wnt/β-catenin signaling pathway (27). Consistently, in this study, we found that silencing *KIF23* in TPC-1 and K1 cells led to the downregulation of Wnt/β-catenin pathway-related proteins, including β-catenin and c-Myc. Consequently, this inhibited the proliferation and migration of PTC cells. Notably, the addition of a Wnt agonist partially rescued the reduced migration abilities of PTC cells caused by *KIF23* knockdown. These findings suggest that KIF23 may contribute to PTC progression by modulating the Wnt/β-catenin signaling pathway.

Mitophagy is a selective form of autophagy that removes damaged mitochondria, thereby maintaining mitochondrial and cellular homeostasis (28, 29). In recent years, mitophagy has gained significant attention for its dual role in tumorigenesis and progression. In the early stages of cancer, mitophagy suppresses tumor growth by eliminating damaged mitochondria, reducing reactive oxygen species (ROS) production, and maintaining cellular homeostasis (30). For example, PINK1 and Parkin, key proteins in the ubiquitin-dependent mitophagy pathway, have been shown to be critical in tumor suppression (31). In KRAS-driven pancreatic cancer, loss of either Parkin or PINK1 increases tumor burden and metastasis, suggesting that these proteins play an early tumor-suppressive role through mitochondrial maintenance (32). However, in advanced tumor stages, mitophagy instead promotes tumor invasion and metastasis by regulating metabolic demands, cell survival, and inflammatory states in the tumor microenvironment (30). For example, mitophagy mediated by PINK1 in lung adenocarcinoma cells and tissues can promote oxidative phosphorylation and redox homeostasis, leading to the development of sustained drug resistance in tumor cells and resulting in poor prognosis (33). In this study, we performed GSEA on transcriptomic data from the TCGA-THCA dataset, revealing significant enrichment of mitophagy. Based on these findings, we hypothesize that KIF23 may influence the invasion and metastasis of PTC cells by regulating mitophagy.

Recent studies have highlighted a close relationship between the Wnt/β-catenin signaling pathway and mitophagy in cellular function and disease progression. For instance, it has been shown that thymoquinone can regulate the Wnt/β-catenin signaling pathway and, through its effects on mitophagy-related mechanisms, influence the growth dynamics of human colorectal HT-29 cells (34). Furthermore, Zhuang *et al.* reported that Escherichia coli infection can activate the Wnt/β-catenin pathway, leading to iron dysregulation and subsequently promoting mitophagy (35). In addition, research has shown that mitophagy, by regulating mitochondrial quality control, plays a crucial role in the development and progression of PTC by influencing energy production, ROS generation, apoptosis, and calcium (Ca^2+^) metabolism (36). In our study, we found that *KIF23* silencing led to increased levels of mitophagy-related markers (Parkin, PINK1, and LC3B). Notably, treatment with a Wnt agonist reversed the increase in mitophagy marker expression caused by *KIF23* knockdown. Moreover, the addition of mitophagy inhibitors partially restored the decrease in PTC cell migration induced by *KIF23* knockdown. These findings suggest that KIF23 may influence PTC development by modulating mitophagy through the Wnt/β-catenin signaling pathway.

However, this study has several limitations. First, the sample size of clinical tissues used for IHC analysis was relatively small, consisting of only 15 cases. A larger sample size is needed to validate our findings. Second, we established a *KIF23* knockdown lentiviral model but did not perform overexpression experiments. To further validate our results, functional assays involving *KIF23* overexpression are necessary. Finally, to gain a deeper understanding of KIF23’s role in PTC progression, we plan to conduct animal studies, such as nude mouse tumorigenesis, to provide more robust theoretical evidence.

In summary, our study found that *KIF23* is significantly elevated in PTC and is closely associated with poor prognosis. Further experiments suggest that KIF23 may regulate mitophagy through the Wnt/β-catenin signaling pathway, thereby affecting the proliferation and migration of PTC cells.

## Supplementary materials









## Declaration of interest

The authors declare that there is no conflict of interest that could be perceived as prejudicing the impartiality of the work reported.

## Funding

This work was supported by grants from the National Natural Science Funds of China (no. 82460168); Jiangxi Provincial Science and Technology Innovation Base Plan – Provincial Clinical Medical Research Center (no. 2020BCG74001); Jiangxi Provincial Science and Technology Innovation Base Construction-Clinical Medical Research Center (no. 20221ZDG02011).

## Author contribution statement

YL was responsible for article design and data analysis. JZ, YC, and MZ helped in manuscript writing. WC and ZH contributed to table making. YD and XH were responsible for figure creation and article layout. JX helped in reviewing and revising manuscripts. All authors contributed to the article and approved the final manuscript.

## Ethical approval and consent to participate

Our study was approved by the Ethics Committee of the First Affiliated Hospital of Nanchang University (Approval No. IIT-2023-384).
