# Supplementary figures and images for: KIF23 silencing suppresses papillary thyroid carcinoma metastasis by regulating mitophagy via Wnt/β-catenin pathway
Source: Endocr Connect. 2025 Oct 24;14(10):e250090. doi: 10.1530/EC-25-0090 (PMC12555028; doi:10.1530/EC-25-0090)

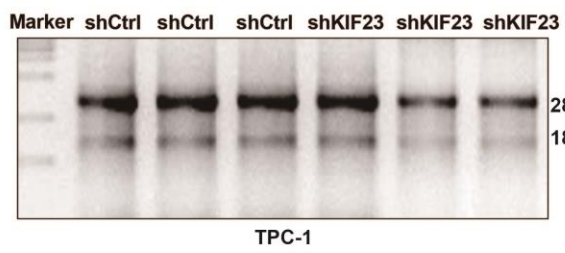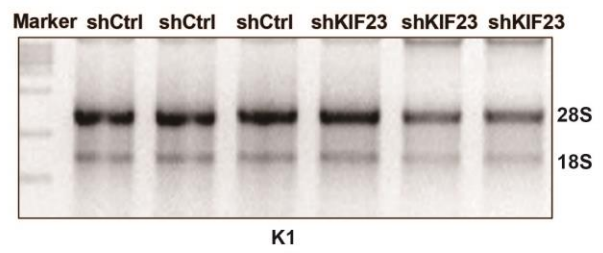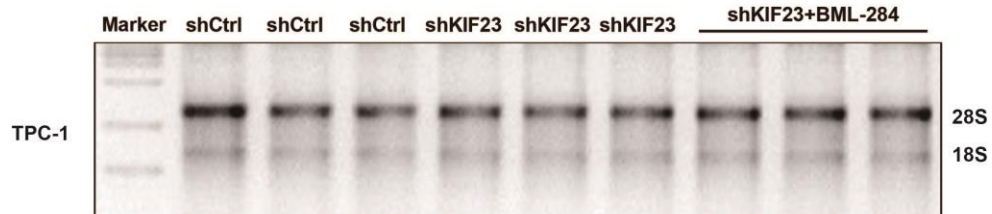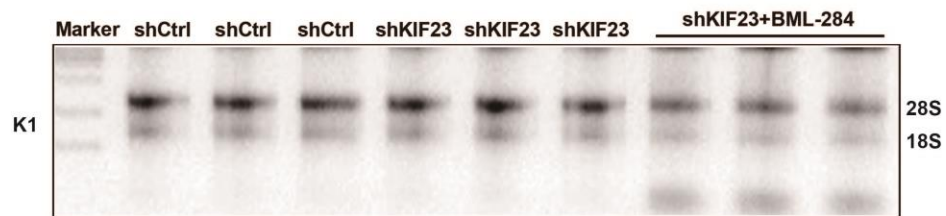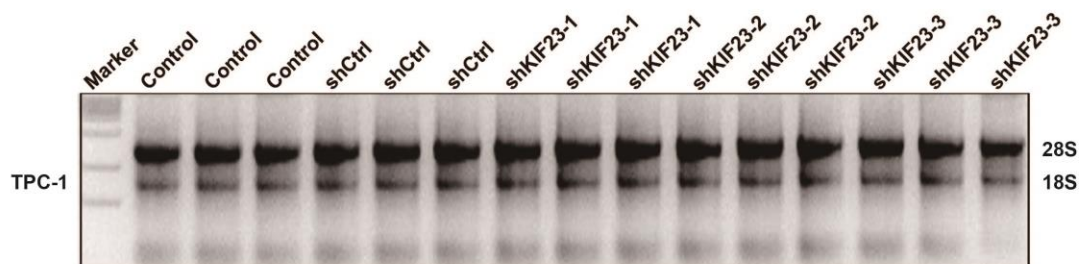

Supplement: Supplementary file 1 [file supplementary_figure_1.pdf]

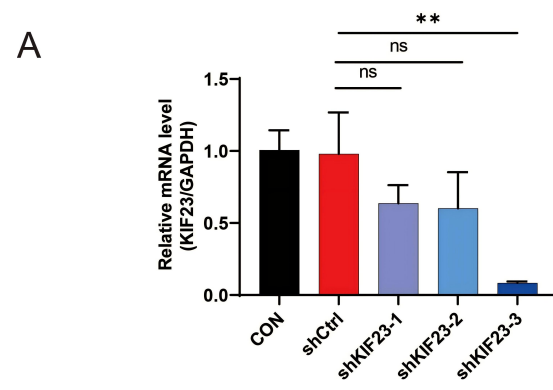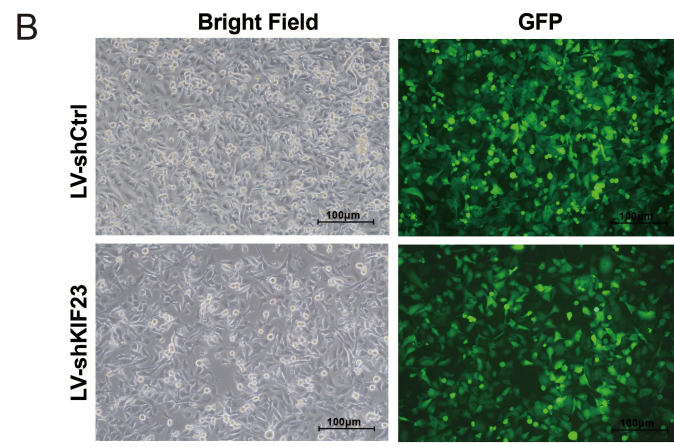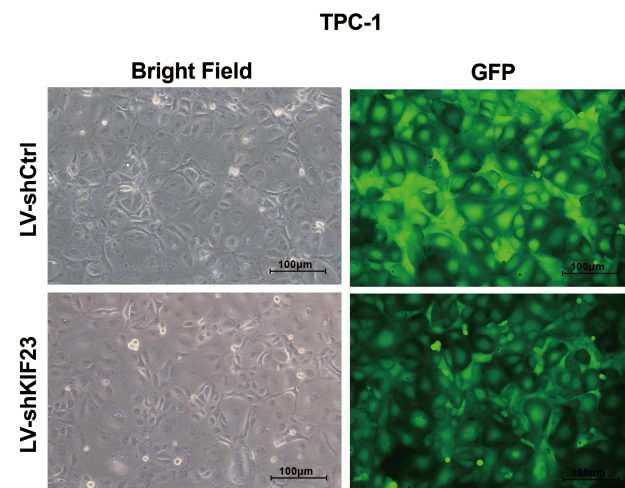

K1

Supplement: Supplementary file 2 [file supplementary_figure_2.pdf]

A

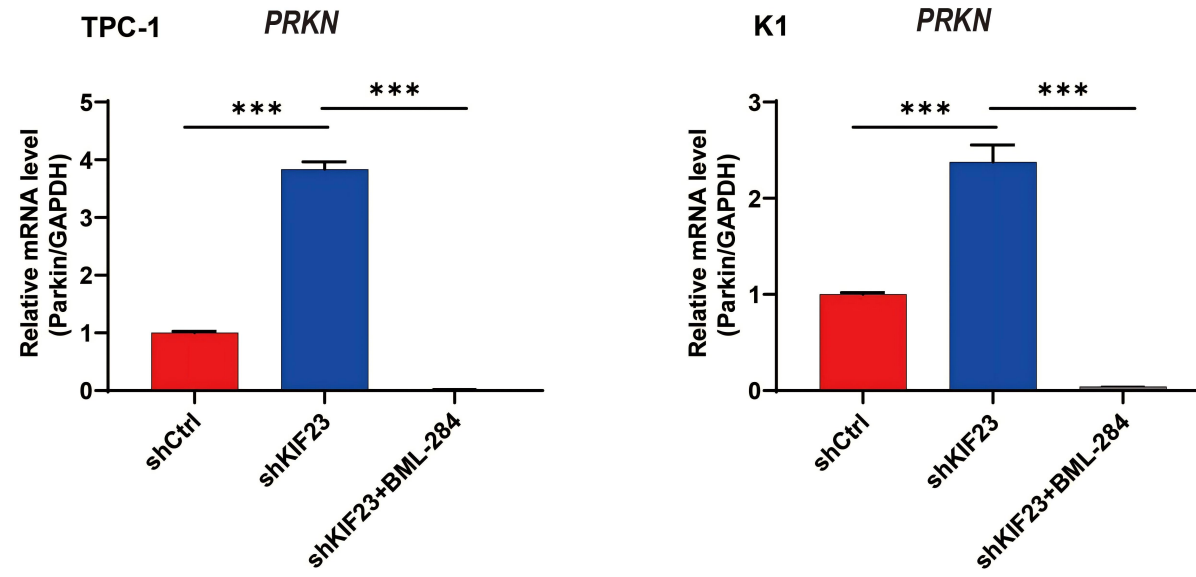

B

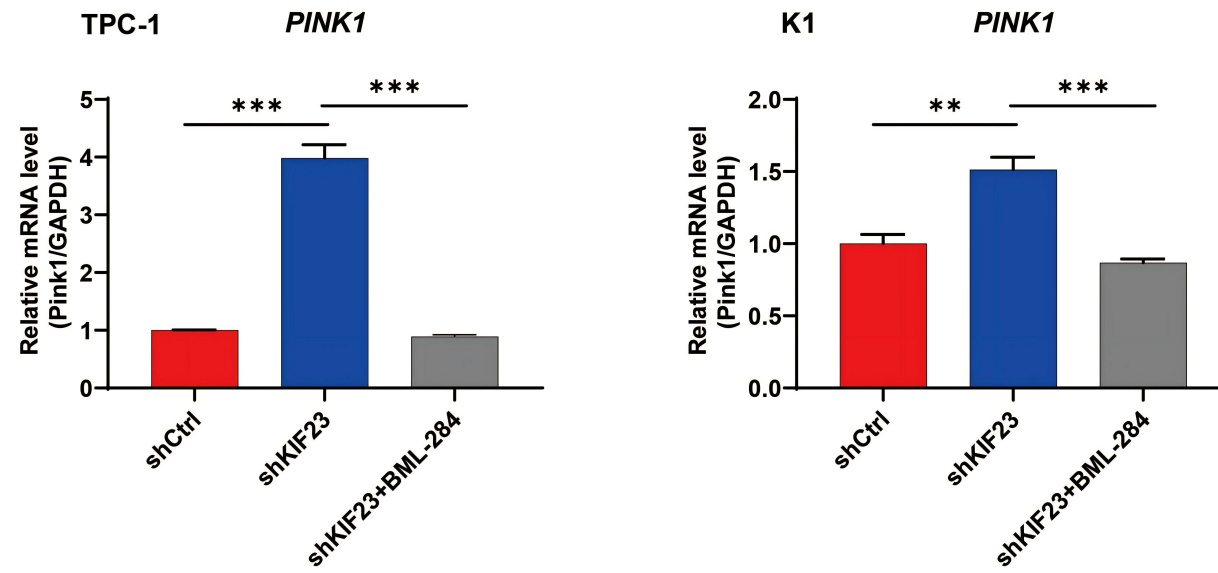

Supplement: Supplementary file 3 [file supplementary_figure_3.pdf]
