# Supplementary material for: KIF23 silencing suppresses papillary thyroid carcinoma metastasis by regulating mitophagy via Wnt/β-catenin pathway
Source: Endocr Connect. 2025 Oct 24;14(10):e250090. doi: 10.1530/EC-25-0090 (PMC12555028; doi:10.1530/EC-25-0090)
Supplement: Supplementary file 4 [file supplementary_tables.pdf]

Primer sequences for target genes

| Primer name | sequence |
| --- | --- |
| Parkin（F） | 5’-GCTGACCAGTTGCGTGTGA-3’ |
| Parkin（R） | 5’-GTGAACAATGCTCTGCTGATCC -3’ |
| PINK1（F） | 5’-CCCAAGCAACTAGCCCCTC-3’ |
| PINK1（R） | 5’-GGCAGCACATCAGGGTAGTC-3’ |
| KIF23（F） | 5’-TGTGGCTAATCCCTTGGTCAA-3’ |
| KIF23（R） | 5’-AGAACCAGTCATTGTGTGAGTTT-3’ |
| GAPDH（F） | 5’-TGACTTCAACAGCGACACCCA-3’ |
| GAPDH（R） | 5’-CACCCTGTTGCTGTAGCCAAA-3’ |

Supplementary table 1

Interference target sequence and shRNA sequence of mRNA for KIF23

| | Sequence |
|---|---|
| (21198)-KD-1 | Target sequence：5’-AACGACATAACTTACGACAAA-3’ |
| | shRNA Sequence：5’-AACGACATAACTTACGACAAActcgagT-TTGTCGTAAGTTATGTCGTT-3’ |
| (21199)-KD-2 | Target sequence：5’-GCGGAAGTGACTCAAGAAGTT-3’ |
| | shRNA Sequence：5’-GCGGAAGTGACTCAAGAAGTTctcgagA-ACTTCTTGAGTCACTTCCGC-3’ |
| (21200)-KD-3 | Target sequence：5’-GGTGGACAATCTGTTCAGTTT-3’ |
| | shRNA Sequence：5’-GGTGGACAATCTGTTCAGTTTctcgagA-AACTGAACAGATTGTCCACC-3’ |

Supplementary table 2
